# Pathological Characterization of Ovarian Cancer Patients Who Underwent Debulking Surgery in Combination With Diaphragmatic Surgery: A Cross-Sectional Study

**DOI:** 10.1097/MD.0000000000002296

**Published:** 2015-12-18

**Authors:** Takeshi Nagai, Hisashi Oshiro, Yasukazu Sagawa, Kentaro Sakamaki, Fumitoshi Terauchi, Toshitaka Nagao

**Affiliations:** From the Department of Anatomic Pathology (TN, HO, TN); Department of Obstetrics and Gynecology, Tokyo Medical University, Tokyo (YS, FT); Department of Biostatics and Epidemiology, Yokohama City University Graduate School of Medicine, Kanagawa, Japan (KS).

## Abstract

Despite exhaustive efforts to detect early-stage ovarian cancers, greater than two-thirds of patients are diagnosed at an advanced stage. Although diaphragmatic metastasis is not rare in advanced ovarian cancer patients and often precludes optimal cytoreductive surgery, little is known about the mechanisms and predictive factors of metastasis to the diaphragm. Thus, as an initial step toward investigating such factors, the present study was conducted to characterize the pathological status of ovarian cancer patients who underwent debulking surgery in combination with diaphragmatic surgery.

This is a retrospective and cross-sectional study of patients who underwent debulking surgery in combination with diaphragmatic surgery at our institution between January 2005 and July 2015. Clinicopathological data were reviewed by board-certified gynecologists, pathologists, and cytopathologists. The rates of various pathological findings were investigated and compared by Fisher exact test between 2 groups: 1 group that was pathologically positive for diaphragmatic metastasis (group A) and another group that was pathologically negative for diaphragmatic metastasis (group B).

Forty-six patients were included: 41 patients pathologically positive and 5 pathologically negative for diaphragmatic metastasis. The rates of metastasis to the lymph node (95.8% vs 20%, *P* = 0.001) and metastasis to the peritoneum except for the diaphragm (97.6% vs 60.0%, *P* = 0.028) were significantly increased in group A compared with group B. However, no significant differences between the 2 groups were found for rates of histological subtypes (high-grade serous or non-high-grade serous), the presence of ascites, the presence of malignant ascites, exposure of cancer cells on the ovarian surface, blood vascular invasion in the primary lesion, and lymphovascular invasion in the primary lesion.

Our study demonstrated that metastasis to the lymph node and nondiaphragmatic metastasis to the peritoneum are significantly associated with metastasis to the diaphragmatic peritoneum, indicating that these factors may be pathological predictors of diaphragmatic metastasis in patients with ovarian cancer. However, as the data available are not sufficient to demonstrate the predictive power of these factors, a further comprehensive, large-scale study should be performed.

## INTRODUCTION

Ovarian cancer remains a serious disease, with an estimated 238,700 newly diagnosed cases and 151,900 deaths in 2012 worldwide.^1^ Despite exhaustive efforts to detect early-stage ovarian cancers, greater than two-thirds of ovarian cancer patients are diagnosed at an advanced stage (International Federation of Gynecology and Obstetrics (FIGO) stage III or IV), resulting in low survival rates (18.6–46.7% 5-year survival rate).^2^ Although women with advanced ovarian cancer have historically been treated with primary debulking surgery followed by platinum- and taxane-based chemotherapy,^3^ the standard management of advanced-stage ovarian cancer remains a subject of debate.^4^

A recent meta-analysis demonstrated that the most important prognostic factor for survival in such patients is the amount of residual tumor after surgery.^5^ The study revealed that each 10% increase in the proportion of patients undergoing cytoreduction without macroscopic residual disease is associated with a significant and independent 2.3-month increase in survival. Therefore, the primary aim in ovarian cancer treatment is to achieve optimal cytoreductive surgery. However, advanced ovarian cancer patients often present with upper abdominal metastases.^6^ In such patients, diaphragmatic metastasis is observed in approximately 40% of cases^6–8^ and in fact precludes optimal cytoreductive surgery in up to 76% of cases.^6^ Nevertheless, little attention has been paid to the mechanisms of diaphragmatic metastasis in ovarian cancer or to the pathological factors predictive of this metastasis.

The diaphragm is one of the widest organs; it separates the thoracic and abdominal cavities, forming a dome-like structure with a very steep slope in the back.^9^ The diaphragm's main functional role is thought to involve breathing movement.^9^ However, the diaphragm also plays another important role by absorbing substances from the abdominal cavity via the lymphatic drainage system.^10^ The diaphragm is situated in the abdomen adjacent to the liver, esophago-gastric junction, inferior vena cava, abdominal aorta, thoracic duct, spleen, adrenal gland, kidney, and pancreas. In combination with ventilatory movement, these anatomical features often make diaphragmatic inspection and operation difficult and time-consuming. Accordingly, some investigators hypothesize that diaphragmatic metastasis may be underestimated during ovarian cancer surgery.^11^

The identification of predictive factors for diaphragmatic metastasis would enable the stratification of patients with regard to the decision of whether to dedicate effort to diaphragmatic investigation during surgery. However, no adequate data are currently available for demonstrating pathological predictors of diaphragmatic metastasis. Thus, as an initial step toward investigating such factors, the present study was conducted to characterize the pathological status of ovarian cancer patients who underwent debulking surgery in combination with diaphragmatic surgery.

## METHODS

This cross-sectional study was approved by our institutional review board (No. 2863). The inclusion criterion was that the patients underwent primary, interval or secondary debulking surgery in combination with diaphragmatic surgery for ovarian carcinomas or carcinosarcomas at Tokyo Medical University Hospital. The exclusion criterion was the lack of available pathological samples, including diaphragmatic materials. The medical records of potentially eligible patients treated from January 2005 to July 2015 were retrospectively and consecutively retrieved from our computerized database. Board-certified gynecologists reviewed patients’ medical charts and investigated information regarding patient age, clinical history, surgical procedures, and administration of chemotherapy for ovarian cancer. Board-certified pathologists evaluated histological samples and diagnosed them according to established criteria^12^ and investigated pathological findings to determine the histological subtypes of the tumors (high-grade serous or non-high-grade serous), exposure of cancer cells on the ovarian surface, metastasis to the peritoneum, and metastasis to the lymph nodes. Elastica-van Gieson staining and immunohistochemistry were performed to evaluate blood vascular or lymphovascular invasion in the primary ovarian lesion using 1 representative formalin-fixed, paraffin-embedded block per case. Immunohistochemistry was performed using antibodies against Von Willebrand factor (F8/86; Dako Japan; Tokyo, Japan) and D2-40 (D2-40; Nichirei Bioscience; Tokyo), a detection kit (Histofine Simple Stain MAX PO, MULTI; Nichirei Bioscience) and an autostainer (Histostainer; Nichirei Bioscience) according to the manufacturer's instructions and a method described in the literature.^13^ For patients with ascites accumulation, cytology was performed to examine whether the ascites contained malignant cells. Board-certified cytopathologists evaluated cytological samples prepared with Papanicolaou, periodic acid-Schiff, Alcian-blue, and Giemsa stains in a blinded manner against histological data and deemed the samples as benign, indeterminate, suspicious for malignancy, or malignant. If the initial judgment was indeterminate or suspicious, cell block samples were prepared to determine whether the specimen was benign or malignant using the rest of the cell sediment in the ascites according to a method described in the literature.^14^ After evaluating the clinicopathological findings, FIGO stages were determined at the time of the debulking and diaphragmatic surgery by both gynecologists and pathologists.

Clinicopathological findings are described by reporting the mean and median values for continuous variables and the frequencies of categorical variables. To compare the rates of various clinicopathological findings between the groups pathologically positive and negative for diaphragmatic metastasis, Student *t* test and the Mann–Whitney *U* test were used for continuous variables, and Fisher exact test was used for categorical variables. Missing values were excluded from the statistical analyses. All statistical analyses were performed using IBM SPSS Statistics 21, and a *P* value of less than 0.05 (2-sided) was considered to indicate statistical significance. If possible, further detailed analysis was performed for statistically significant pathological variables.

## RESULTS

**FIGURE 1:**
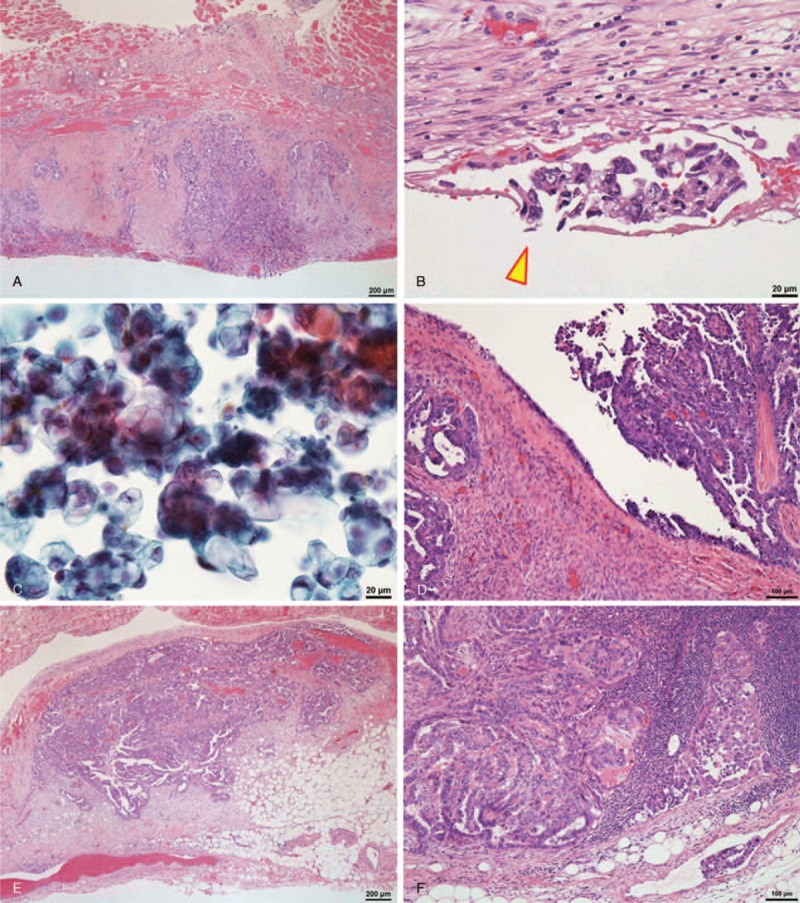
A representative case of high-grade serous carcinoma. A, Carcinoma exhibiting metastasis to the diaphragmatic peritoneum (hematoxylin and eosin stain). B, Carcinoma involving a stoma (arrowhead) of the diaphragmatic peritoneum (hematoxylin and eosin stain). C, Carcinoma cells in the ascites detected by cytological examination (Papanicolaou stain). D, Carcinoma presented on the ovarian surface (hematoxylin and eosin stain). E, Carcinoma showing metastasis to the mesentery (hematoxylin and eosin stain). F, Carcinoma metastasizing to the external iliac lymph node (hematoxylin and eosin stain).

Between Jan 2005 and Jul 2015, 267 patients underwent surgery for ovarian carcinomas or carcinosarcomas at our institution; among them, 46 patients fulfilled our inclusion criterion, and none were eliminated based on our exclusion criterion. The clinical characteristics of the 46 patients with ovarian cancer who underwent debulking surgery in combination with diaphragmatic surgery are summarized in Table 1. Our study population included 5 patients without metastasis to the diaphragmatic peritoneum and 41 patients with metastasis to the diaphragmatic peritoneum (Figure 1A and B). No significant differences were noted among the 2 groups regarding patient age, type of debulking surgery, type of diaphragmatic surgery, site of diaphragmatic surgery, or chemotherapy. However, significant differences were observed for FIGO stages (*P* = 0.001) and the laterality of ovarian cancer between the 2 groups (*P* = 0.026).

**TABLE 1:**
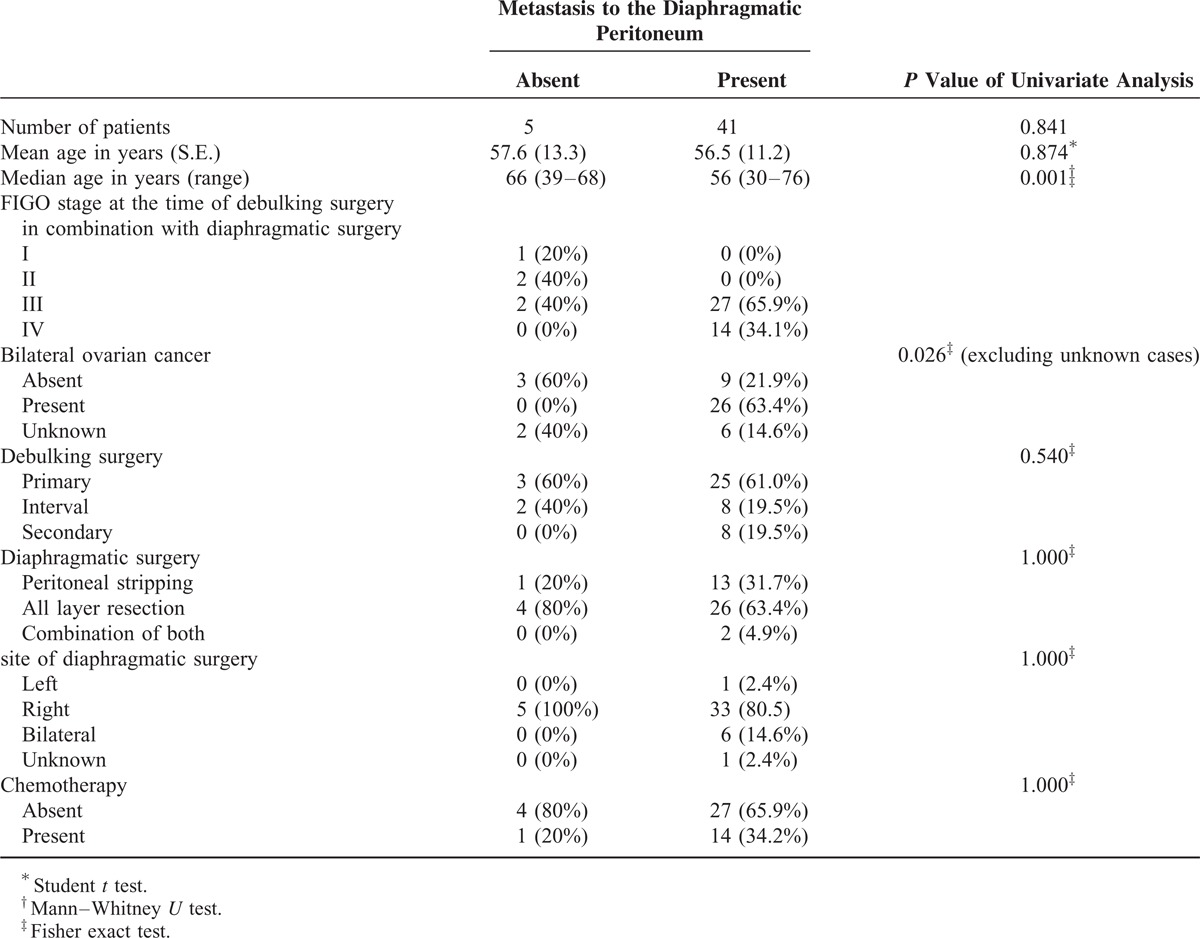
Clinical Characteristics of 46 Patients With Ovarian Cancers Who Underwent Debulking Surgery in Combination With Diaphragmatic Surgery

The pathological characteristics of the 46 patients are summarized in Table 2. In the group that was negative for diaphragmatic metastasis, 3 cases were high-grade serous carcinomas, 5 cases had ascites (3 of which were found to have malignant ascites), 3 cases exhibited exposure of cancer cells on the ovarian surface, 3 cases exhibited metastasis to other peritoneal locations other than the diaphragm, and 1 case exhibited metastasis to the lymph nodes. In the group that was positive for diaphragmatic metastasis, 30 cases were high-grade serous carcinomas, 39 cases had ascites (35 of which were found to have malignant ascites (Figure 1C), with 2 cases requiring cell block samples for final judgment), 2 cases did not have ascites (1 of which had malignant cells, as identified by peritoneal washing cytology), 28 cases demonstrated exposure of cancer cells on the ovarian surface (Figure 1D), 40 cases exhibited metastasis to other peritoneal locations in addition to the diaphragm (Figure 1E), and 23 cases exhibited metastasis to the lymph nodes (Figure 1F).

**TABLE 2:**
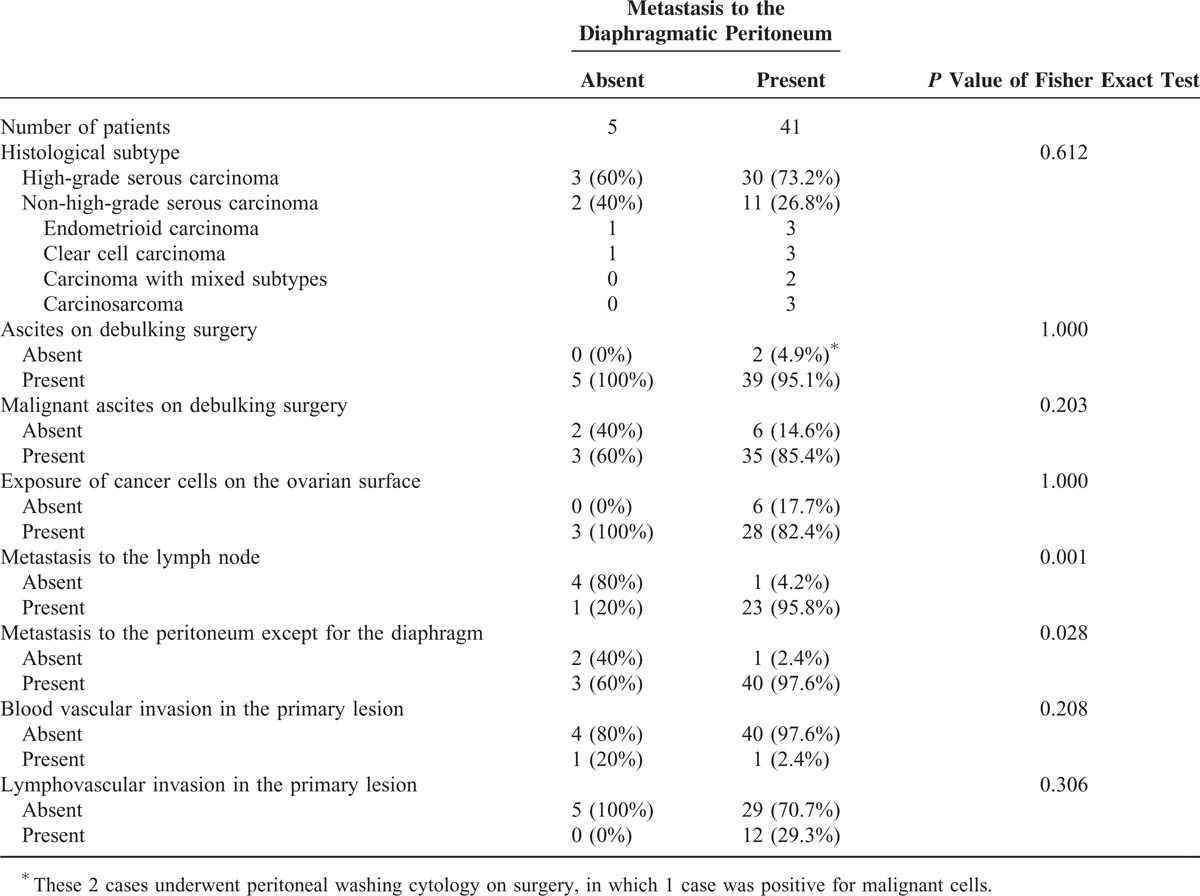
Pathological Characteristics of 46 Patients With Ovarian Cancers Who Underwent Debulking Surgery in Combination With Diaphragmatic Surgery

The rates of metastasis to the lymph nodes (95.8% vs 20%, *P* = 0.001) and nondiaphragmatic peritoneal metastasis (97.6% vs 60.0%, *P* = 0.028) were significantly increased in the group pathologically positive for diaphragmatic metastasis compared with the negative group. In addition, the rates of histological subtype (high-grade serous or non-high-grade serous) (73.2% vs 60.0%, *P* = 0.612), the presence of ascites (95.1% vs 100%, *P* = 1.000), the presence of malignant ascites (85.4% vs 60.0%, *P* = 0.203), the exposure of cancer cells on the ovarian surface (82.2% vs 100%, *P* = 1.000), blood vascular invasion in the primary lesion (2.4% vs 20.0%, *P* = 0.208), and lymphovascular invasion in the primary lesion (29.3% vs 0%, *P* = 0.306) did not differ significantly between the 2 groups.

**FIGURE 2:**
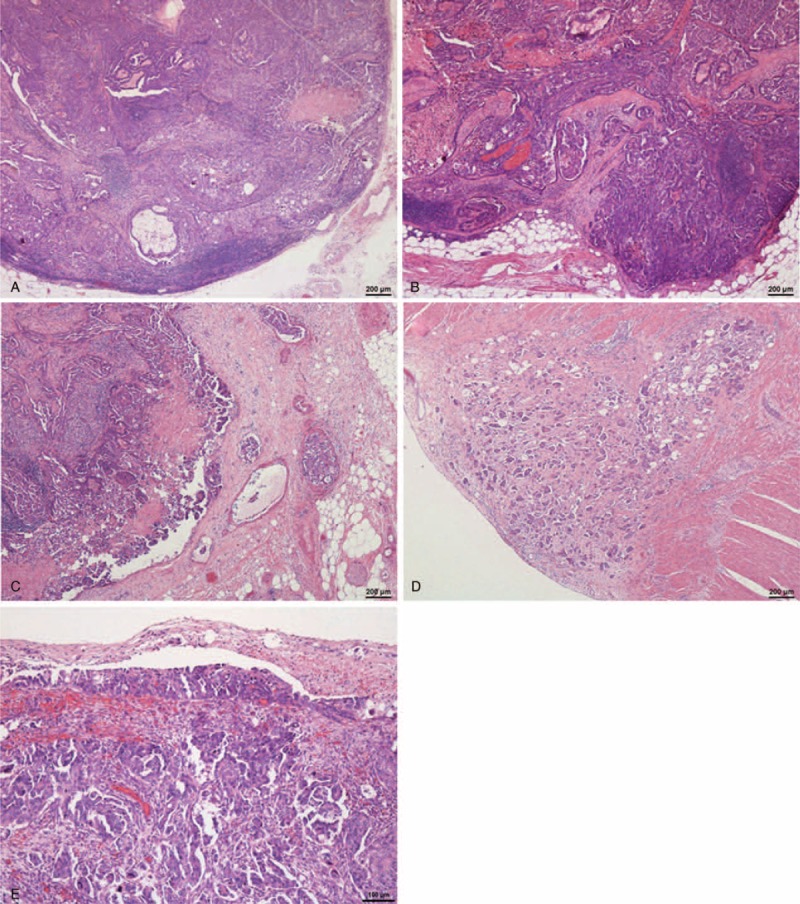
A representative case of high-grade serous carcinoma. A, Carcinoma exhibiting metastasis to the 326B lymph node (hematoxylin and eosin stain). B, Carcinoma exhibiting metastasis to the internal iliac lymph node (hematoxylin and eosin stain). C, Carcinoma exhibiting metastasis to the greater omentum. D, Carcinoma exhibiting metastasis to the serosa of the colon (hematoxylin and eosin stain). E, Carcinoma exhibiting metastasis to the Douglas pouch (hematoxylin and eosin stain).

Further detailed analysis revealed significant increases in the group that was positive for diaphragmatic metastasis for the rates of metastasis to the 326B (abdominal para-aortic) lymph node (89.5% vs 20.0%, *P* = 0.006) (Figure 2A), metastasis to lymph nodes other than 326B (73.9% vs 20.0%, *P* = 0.041) (Figure 2B), metastasis to the greater omentum (94.1% vs 25.0%, *P* = 0.005) (Figure 2C), and metastasis to the peritoneum except for the diaphragm, Douglas pouch/uterus (Figure 2D) and greater omentum (90.0% vs 40.0%, *P* = 0.021) (Figure 2D) (Table 3). In addition, the rate of metastasis to the Douglas pouch/uterus (Figure 2E) did not significantly differ between the 2 groups (*P* = 0.120).

**TABLE 3:**
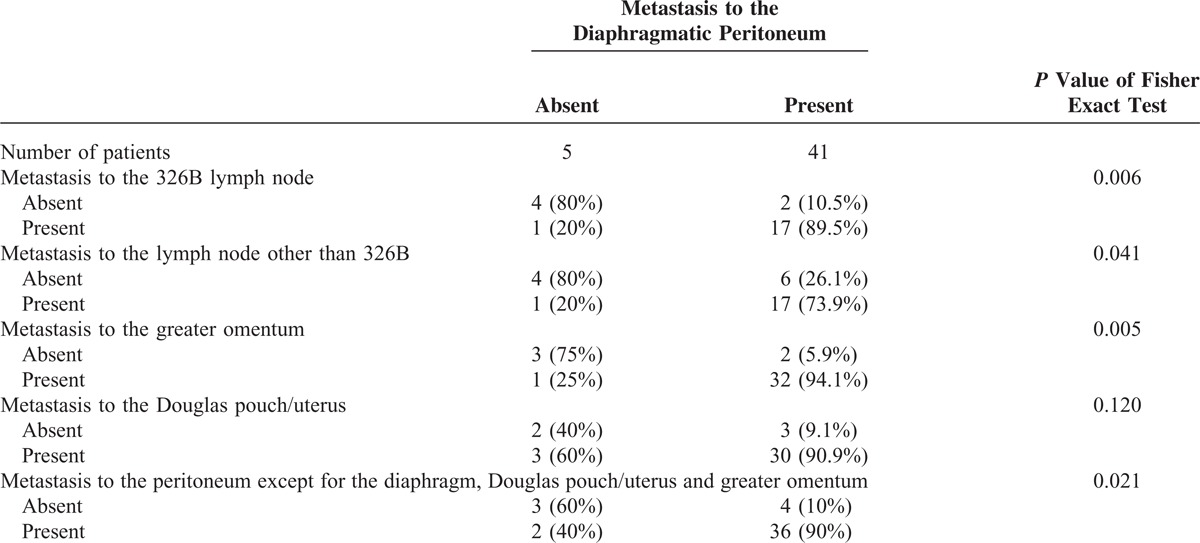
Pathological Detailed Analysis of Lymph Nodes and Peritoneal Regions of Patients With Cancers

## DISCUSSION

The main result of this study was that rates of metastasis to the lymph nodes and metastasis to the peritoneum except for the diaphragm were significantly increased in the group that was pathologically positive for diaphragmatic metastasis compared with the group that was pathologically negative for diaphragmatic metastasis.

Metastasis is thought to occur via transcoelomic, lymphogenous or hematogenous routes or a combination of these routes.^15^ For ovarian cancer, a prevailing notion is that cancer metastasis to the peritoneum mainly occurs via the transcoelomic route.^16–19^ Based on this hypothesis, metastasis to the diaphragm might be predicted by the presence of malignant ascites or by the exposure of cancer cells on the ovarian surface. However, our results did not demonstrate significant differences in the rates of these factors between the 2 groups studied. Notably, despite an absence of malignant ascites, there were a few cases exhibiting metastasis to the diaphragmatic peritoneum. In addition, a non-negligible number of cases demonstrated no metastasis to the diaphragmatic peritoneum despite the presence of malignant ascites and the exposure of cancer cells on the ovarian surface. Experimental studies have demonstrated that a large number of cancer cells are required to achieve dissemination via the transcoelomic route, presumably 2 × 10^6^ cells in an immunologically normal hamster^20^ and 1 × 10^5^ to 10^6^ cells in an immunologically normal rabbit,^21^ which are sufficient numbers to detect by routine cytological examination, as performed in the present study. Clearly, the presence of cancer cells itself may not be sufficient to achieve transcoelomic metastasis. Indeed, metastasis likely requires a particular microenvironment, such as a fluid that efficiently supports cancer cell growth as well as migration to and grafting in distant peritoneal regions.^22^ Alternatively, in the absence of such a fluid, a metastatic lesion in the peritoneum, such as in the greater omentum, might result in contact metastasis to the diaphragmatic peritoneum.

Asai-Sato et al reported that the sensitivity of peritoneal swabbing cytology for the detection of neoplastic cells in the peritoneal cavity is only 40% in ovarian carcinomas/borderline tumors.^23^ Their data indicate that the rate of pathological exposure of cancer cells on the peritoneal metastatic lesion is lower than clinically expected in cases with peritoneal dissemination of ovarian cancers, and this lower detection rate may be because cancer cells immediately migrate to the subserosal layer following attachment to the serosal membrane. Nonetheless, adequate data are not available to support this assumption. The results of the present study contradict the hypothesis that the diaphragmatic metastasis of ovarian cancer mainly occurs via the transcoelomic route. Interestingly, several studies suggest that accumulation of malignant ascites is not likely to be a predominant cause of diaphragmatic metastasis but rather a result of diaphragmatic metastasis involving a considerable degree of lymphatic obstruction.^24–26^ However, the causal relationship has yet to be verified.

The second possibility is the lymphogenous metastatic route. The rich lymphatic network equipped with lymphatic stomata distinguishes the diaphragmatic peritoneum from other peritoneal regions.^10,27,28^ Under physiological conditions, the lymphatic drainage system plays an important role in the egress of fluid from the abdominal cavity via the lymphatic stomata in the diaphragmatic peritoneum.^29–31^ The lymphatic vessels issuing from the posterior part of the diaphragm travel backward across the crura and directly connect with the origin of the thoracic duct or indirectly connect with the thoracic duct via intercalation of the upper para-aortic lymph node.^32^ Recent studies have also demonstrated that the thoracic duct plays a specific role in lymphatic drainage from the abdominal cavity of quadrupeds.^33–35^ These reports suggest an integral role of the upper para-aortic lymphatic system in bipeds, whereby a certain volume of diaphragmatic lymphatic fluid is thought to drain toward the thoracic duct. In addition, the lymphatic spread of ovarian cancer is thought to occur mainly via pelvic and para-aortic pathways in an antegrade manner.^36^ It is interesting to note that metastasis to the 326B (abdominal para-aortic) lymph node was significantly associated with metastasis to the diaphragmatic peritoneum (*P* = 0.006). Lymphogenous metastasis appears to contradict the concept of normal lymphatic flow. However, studies have shown that lymphatic pathways exist from the para-aortic lymph node to the diaphragm,^37,38^ that lymphatic flows in the diaphragm are multidirectional in nature,^39,40^ and that the diaphragmatic lymphatic system possesses a network organized into a series of confluent vessels that can functionally adapt to regional drainage requirements by recruiting lymphatics.^41^ In addition, changes in lymphatic dynamics caused by primary surgery or cancer dissemination might affect the direction of peritoneal or retroperitoneal lymphatic flow, directing more flow toward the diaphragm.^42^ The hypothesis of the lymphogenous metastatic pathway is also supported by the concept of the retrograde or rerouting lymphatic spread of cancer cells.^43–46^ Alternatively, it is possible that cancer cells spread from the metastasized peritoneum to the diaphragm via peritoneal lymphatics with or without the intercalation of lymph nodes, as suggested in an experimental study.^47^

The last possibility is the hematogenous route. If diaphragmatic metastasis occurs via this route, a significant number of metastatic lesions should be observed in extra-abdominal organs such as the lung or brain. However, no such signs were detected by imaging studies prior to debulking surgery in our cases. Some investigators suggest that cancer spread can occur via lympho-venous shunts.^48,49^ However, such shunts are seldom demonstrated by lymphangiography and then only in cases with an abnormal increase in intralymphatic pressure resulting from obstruction of the lymph flow.^48,49^ This obstruction may be due to lymphatic metastases, previous surgery, or inflammatory or infectious diseases.^48,49^ Additional research is needed to clarify whether lympho-venous shunts contribute to metastasis to the diaphragmatic peritoneum.

The present study has several limitations. First, the study was performed retrospectively using surgical cases. As organs that were suspected to have metastases by gross examination were more likely to be removed by surgeons and subjected to pathological examination, the result was a small number of cases exhibiting no metastasis to the diaphragm. Moreover, although the mean age (56.5–57.6 years vs 57.6 years) and predominance of serous carcinoma histology (71.7% vs 52.4%) of our ovarian cancer patient series were roughly equal to those based on the FIGO 26th annual report, the proportions of FIGO stages in our patient series were lower in stage I (2.1% vs 35.5%) and higher in stages III (63.0% vs 44.6%) and IV (30.4% vs 11.7%) compared with those in the report,^2^ which might explain the higher rate of bilateral ovarian cancer in our patient series compared with that based on a study from a pooled German national dataset (56% vs 26.0%).^50^ These phenomena could be a source of bias in our study. Second, given the relatively small number of cases, it was difficult to calculate the odds ratio and perform logistic regression analysis. Third, as cases were retrieved from only 1 institution, it may be difficult to generalize the results. However, to our knowledge, this study is the first attempt to investigate the pathological status of ovarian cancer patients with and without diaphragmatic metastasis, and we believe that the results provide important information for future studies specifically designed to investigate the mechanisms of the diaphragmatic metastasis of ovarian cancers.

In conclusion, the present study demonstrated that metastasis to the lymph nodes and metastasis to peritoneal sites other than the diaphragm are significantly associated with metastasis to the diaphragmatic peritoneum, indicating that these factors may be pathological predictors of metastasis to the diaphragmatic peritoneum in patients with ovarian cancer. However, further comprehensive, large-scale surveys are needed to elucidate robust predictive factors and mechanisms of ovarian cancer diaphragmatic metastasis.
